# Analysis of Chagas disease vectors occurrence data: the Argentinean triatomine species database

**DOI:** 10.3897/BDJ.8.e58076

**Published:** 2020-11-12

**Authors:** Soledad Ceccarelli, Agustín Balsalobre, Maria Eugenia Cano, Delmi Canale, Patricia Lobbia, Raúl Stariolo, Jorge Eduardo Rabinovich, Gerardo Anibal Marti

**Affiliations:** 1 Centro de Estudios Parasitológicos y de Vectores (CEPAVE CONICET-CCT La Plata-UNLP), La Plata, Argentina Centro de Estudios Parasitológicos y de Vectores (CEPAVE CONICET-CCT La Plata-UNLP) La Plata Argentina; 2 Centro de Referencia de Vectores (CeReVe), Coordinación Nacional de Vectores, Ministerio de Salud de la Nación, Santa Maria de Punilla, Argentina Centro de Referencia de Vectores (CeReVe), Coordinación Nacional de Vectores, Ministerio de Salud de la Nación Santa Maria de Punilla Argentina

**Keywords:** Chagas, Triatominae, database, temporal information, geographic distributions, species richness, habitat

## Abstract

**Background:**

Chagas disease is a neglected tropical disease and *Trypanosoma cruzi* (its etiological agent) is mainly transmitted by triatomines (Hemiptera: Reduviidae). All triatomine species are considered as potential vectors; thus, their geographic distribution and habitat information should be a fundamental guide for the surveillance and control of Chagas disease. Currently, of the 137 species distributed in the Americas (Justi and Galvão 2017), 17 species are cited for Argentina: *Panstrongylus geniculatus*, *P. guentheri*, *P. megistus*, *P. rufotuberculatus*, *Psammolestes coreodes*, *Triatoma breyeri*, *T. delpontei*, *T. eratyrusiformis*, *T. garciabesi*, *T. guasayana*, *T. infestans*, *T. limai*, *T. patagonica*, *T. platensis*, *T. rubrofasciata*, *T. rubrovaria* and *T. sordida*. Almost 20 years have passed since the publication of the “Atlas of the Triatominae” by Carcavallo et al. (1998) and no work has been done to provide an updated complete integration and analysis of the existing information for Argentinean triatomine species. Here we provide a detailed temporal, spatial and ecological analysis of updated occurrence data for triatomines present in Argentina.

**New information:**

This is the first database of the 17 triatomine species present in Argentina (15917 records), with a critical analysis of the temporal, spatial and ecological characteristics of 9788 records. The information spans the last 100 years (1918–2019) and it was mostly obtained from the DataTri database and from the Argentinean Vector Reference Center. As 70% of the occurrences corresponded to the last 20 years, the information was split into two broad periods (pre-2000 and post-2000). Occurrence data for most species show distribution range contractions, which, from the pre-2000 to post-2000 period, became restricted mainly to the dry and humid Chaco ecoregions. Concurrently, the highest species richness foci occurred within those ecoregions. The species *T. infestans*, *T. sordida*, *T. garciabesi* and *T. guasayana* mostly colonise human dwelling habitats. This study provides the most comprehensive picture available for Argentinean triatomine species and we hope that any knowledge gaps will encourage others to keep this information updated to assist health policy-makers to make decisions based on the best evidence.

## Introduction

Chagas disease is a public health problem in the Americas and elsewhere (World Health Organization 2017) that has *Trypanosoma cruzi* as its etiological agent. Despite the existence of other infection routes (blood transfusions, laboratory accidents, congenital transmission and orally via contaminated food), vectorial transmission by Triatominae (Hemiptera: Reduviidae) is currently the main route of infection. Thus, up-to-date information on the geographic occurrence of triatomine species becomes important and necessary to understand the epidemiological and control aspects of this disease. Although all Neotropical triatomine species (around 150) are considered as potential vectors, about 70 species have been found to be naturally infected with this parasite (Galvão and Justi 2015) and their geographic distribution information should be a fundamental guide for the surveillance and control of Chagas disease. Some studies that integrated the geographic information of Argentinean triatomines (Abalos and Wygodzinsky 1951, Martinez and Cichero 1972, Carcavallo et al. 1998) are still used as bibliographical references, despite being 25-70 years old. Almost 20 years have passed since the publication of the “Atlas of the Triatominae” by Carcavallo et al. (1998) and no work has been done to provide an updated complete integration and analysis of the existing geographic information for Argentinean triatomine species, as in other Latin American countries, such as Brazil (Galvão 2014), Mexico (Salazar-Schettino et al. 2010, Ramsey et al. 2015), Colombia (Guhl et al. 2007), French Guiana (Bérenger et al. 2009), Suriname (Hiwat 2014), Peru (Chávez 2006) and Venezuela (Cazorla-Perfetti and Nieves-Blanco 2010). Currently, of the 137 species distributed in the Americans (Justi and Galvão 2017), 17 species are cited for Argentina: *Panstrongylus geniculatus*, *P. guentheri*, *P. megistus*, *P. rufotuberculatus*, *Psammolestes coreodes*, *Triatoma breyeri*, *T. delpontei*, *T. eratyrusiformis*, *T. garciabesi*, *T. guasayana*, *T. infestans*, *T. limai*, *T. patagonica*, *T. platensis*, *T. rubrofasciata*, *T. rubrovaria* and *T. sordida*. For some of these species, their entire geographic range is circumscribed to Argentina, while others extend their geographic distribution to neighbouring countries, such as Bolivia, Brazil, Chile, Paraguay and Uruguay and there are even cases of widely-distributed species, such as *P. geniculatus* or *P. rufotuberculatus*, whose distribution range reaches Central America and Mexico, respectively (Carcavallo et al. 1998, Ramsey et al. 2015).

The Wallacean shortfall (Hortal et al. 2015) refers to the lack of knowledge about the geographic distribution of species (Lomolino 2004) and it is intimately connected with temporal and spatial variations in surveying effort (Boakes et al. 2010. At smaller scale (e.g. countries), the Wallacean shortfall becomes more marked, as increasingly precise information about geographic distribution is required (Riddle et al. 2011). Therefore, primary data, i.e. dated records of species occurrences, are the best kind of data, preferable to summaries done at coarser resolutions or that may be missing attributes attached to the original record. In the case of vector species relevant to some vector-borne diseases, there are some initiatives that have compiled vector occurrence data (e.g. mosquitoes (Kraemer et al. 2015) or sandflies (Pigott et al. 2014)), thus providing geographic information that enables policy-makers to make evidence-based decisions. Other vector species are often sparsely recorded (Hay et al. 2013) and there are few globally-comprehensive sets of compiled primary data. Moreover, data on the geographic distribution of vector species is not always publicly available and details of sampling biases or occurrence validation are frequently difficult to find.

In the case of Chagas disease, updated data about current geographic distribution of triatomines are essential resources for the development of strategies to control vector transmission. Knowledge about triatomine habitats (i.e. domicile, peridomicile, sylvatic), as well as their synanthropic behaviour, are epidemiologically relevant since the risk of contracting Chagas disease by vectorial transmission depends mainly on the presence of the triatomines inside human dwellings. Recently, Ceccarelli et al. (2018) published an updated occurrence database for 135 American triatomine species (DataTri) including Argentinean species records. Additionally, the Argentinean Ministry of Health (AMH), through two of its research institutions (‘Centro de Referencia de Vectores’ and ‘Centro Nacional de Diagnóstico e Investigación en Endemoepidemias’, CeReVe and CeNDIE/ANLIS, respectively, after their Spanish acronyms), has also been compiling triatomine occurrence data, based on unpublished entomological reports. Furthermore, triatomine geographic information from a citizen science-based project (GeoVin) is being collected through a mobile application developed by the triatomines laboratory at CEPAVE (CONICET, UNLP).

## General description

### Purpose

The aim of this work is to develop a detailed temporal, spatial and ecological analysis of updated occurrence data for Argentinean triatomines, obtained from the above-mentioned data sources (DataTri, AMH and GeoVin), to achieve the following main goals: (a) provide a temporal description of the collected data, (b) update and refine the current knowledge of geographic distributions for triatomines in Argentina, (c) describe the diversity patterns of Argentinean triatomine species and (d) analyse, categorise and classify triatomine species according to the habitat where they occur.

## Project description

### Title

Triatomine Database Strengthening ("Fortalecimiento de la Base de Datos de Triatominos")

### Personnel

Data gathering and final dataset building was conducted over five years under the responsibility of Soledad Ceccarelli, Agustín Balsalobre, Maria Eugenia Cano, Maria Eugenia Vicente, Paula Medone, Jorge E. Rabinovich and Gerardo A. Marti from the Centro de Estudios Parasitológicos y de Vectores (CEPAVE CONICET-CCT La Plata-UNLP). Delmi Canale, Patricia Lobbia and Raúl Stariolo from the Centro de Referencia de Vectores (CeReVe - Coordinación Nacional de Vectores, Ministerio de Salud de la Nación) provided triatomine occurrence data, based on unpublished entomological reports. Additional help and/or occurrence data were provided by many other colleagues throughout the years.

### Study area description

The geographic area associated to the dataset encompasses southern Argentina and Chile to southern Mexico. The temporal coverage of the dataset spans the period from 1918 to 2019, while the taxonomic coverage includes the 17 species currently cited for Argentina.

### Design description

The main goal of the project was based on: i) to improve the quality of existing data in line with established standards, ii) to refine the current knowledge of geographic distributions for triatomines in Argentina and iii) to provide a public geodatabase with updated occurrence data for triatomine species present in Argentina, based on accurately georeferenced locations (Chapman and Wieczorek 2006).

### Funding

Ministerio de Ciencia y Técnica de la Nación (MinCyT) throughout the Sistema Nacional de Datos Biológicos (SNDB).

## Sampling methods

### Study extent

The methods applied to gather, georeference and validate the Argentinean triatomine species occurrences to build the dataset are described in detail in Ceccarelli et al. (2018); however, here we provide a brief overview.

### Step description

Data were compiled from three main data sources: (1) DataTri (the American triatomine database) (Ceccarelli et al. 2018), a compilation of triatomine occurrences and complementary ecological data representing the most complete and updated database available on triatomine species at a continental scale. It was assembled by collecting the records of triatomine species published from 1904 to 2017, spanning all American countries with triatomine presence. The georeferenced records were obtained from published literature, personal fieldwork and data provided by colleagues; (2) CeReVe (Centro de Referencia de Vectores - Argentinean Ministry of Health – AMH) and CeNDIE/ANLIS (Centro Nacional de Diagnóstico e Investigación en Endemoepidemias - Reference Center for the Diagnosis of Endemoepidemics, also part of AMH), based on records compiled from entomological surveys. CeReVe has the most extensive collection of triatomine occurrence records in Argentina; every year for the last 20 years, the triatomines collected during the spraying campaigns for vector control organised by the Chagas Disease Control Program in different provinces of Argentina, have been taken to CeReVe for taxonomic determination, recorded and curated. When two or more triatomine specimens were collected from different houses at the same locality and on the same date, individuals of the same species were pooled as a single record; (3) GeoVin (www.geovin.com.ar), a citizen science project developed by researchers at CEPAVE and ILPLA (CONICET-Argentinean National Research and Scientific Council and UNLP-National University of La Plata). This project started in 2018 to gather geographic information on triatomines of Argentina through citizen participation using a public and free application; users report bug findings that are georeferenced using their mobile phone GPS. Occurrence data (photos and geographic coordinates) sent online are stored in a centralised server and then data validation is done by researchers of the Triatomine Laboratory at CEPAVE. Once the data are validated, they are automatically integrated into the GeoVin occurrence dataset. Data from 2018 and 2019 were considered to make up this dataset.

**Analysis of the Argentinean triatomine occurrence data**

The analysis of the Argentinean triatomine occurrence data was based upon a three-staged approach: i) temporal, ii) geographic and iii) habitat type.

**Temporal pattern.** To analyse the temporal pattern, the information was taken from each record and classified in 7-year intervals (year represents the year of collection) using only the records with associated information on year or period of years. If records from the published literature did not provide a collection date, the period (pre-2000 or post-2000) to which the publication belonged was assigned.

**Geographic distributions and species richness pattern.** We analysed the occurrence distributions and richness patterns of triatomine species. First, to obtain an overall view of the geographic information for each triatomine species, its occurrence data were mapped over areas representing ecoregions (Burkart et al. 1999) using QGIS software (QGIS Development Team 2018). The geographic distributions of the species *T. garciabesi* and *T. sordida* required particular consideration because their taxonomic classification in certain areas is currently under debate (Gurgel-Gonçalves et al. 2010, Panzera et al. 2015); thus, records belonging to both species that were within the area involving eastern Jujuy and Salta Provinces, northern Santiago del Estero Province and Western Chaco and Formosa Provinces were considered controversial and were classified as ‘*T. garciabesi-T. sordida*’. Records that we considered as having doubtful geographic information or those that were the result of passive transport (i.e. carried by humans or in wood and brick shipments), were classified as ‘questionable information’ and denoted with red stars on the geographic maps of each species. Then, to obtain a pattern of species richness for triatomines of Argentina, the occurrence data (points) of each species from the last 20 years were overlaid by a regular grid of 0.25° (longitude-latitude cells) resolution; from this grid, a presence-absence matrix was built containing all species (columns) and sites/grid cells (rows). In this matrix, the species richness of each cell is represented by the number of different species occurring in that cell. The final species richness values per cell were exported as a raster file and then mapped with the QGIS software (QGIS Development Team 2018) to show the biodiversity pattern maps. Species richness estimates were carried out in the R statistical language (R Core Team 2019), using the ‘maptools’ (Bivand and Lewin-Koh 2018) and ‘letsR’ (Vilela and Villalobos 2015) R packages. The R codes used to calculate species richness estimates from the datasets are available on GitHub (https://github.com/solnqnlp/Triatominae_SpRichness).

**Habitat type.** We classified each triatomine species according to the habitat type where triatomines are found. Although triatomine habitat types are usually classified as belonging to three general environment categories (inside human dwellings or domicile, around human dwellings or peridomicile and natural environment or sylvatic habitat); here we opted for a domiciliation/intrusion level habitat classification, based upon the one proposed by Waleckx et al. (2015). To that end, some particular considerations had to be taken into account: i) only records from the last 20 years were used, ii) the number of records for each species in each basic habitat type (domicile, peridomicile and sylvatic) were assessed from the database, iii) the presence/absence of immature stages in a determined habitat -beyond the presence of adults- was also assessed from the database to determine if a species had the capacity to establish colonies in each habitat; iv) if a record had a mix of habitats, it was duplicated as two records (e.g. habitat information expressed as “Domicile-Peridomicile”, was considered as two records, one as “Domicile” and the other as “Peridomicile”); and v) ‘*T. garciabesi-T. sordida*’ records were included in both the *T. garciabesi* and *T. sordida* datasets. Finally, records with no habitat information were considered as not available (NA).

## Geographic coverage

### Description

The geographic area associated to the dataset encompasses southern Argentina and Chile to southern Mexico.

### Coordinates

-55.777 and 23.241 Latitude; -97.383 and -33.047 Longitude.

## Taxonomic coverage

### Description

The following taxonomic units were identified: three genera (*Panstrongylus*, *Psammolestes* and *Triatoma*) and 17 species: *P. geniculatus*, *P. guentheri*, *P. megistus*, *P. rufotuberculatus*, *Ps. coreodes*, *T. breyeri*, *T. delpontei*, *T. eratyrusiformis*, *T. garciabesi*, *T. guasayana*, *T. infestans*, *T. limai*, *T. patagonica*, *T. platensis*, *T. rubrofasciata*, *T. rubrovaria* and *T. sordida*.

## Traits coverage

We compiled a total of 15917 occurrence data for the entire geographic range of the triatomine species present in Argentina; of those, 9788 records corresponded to exclusively Argentinean distributions (Ceccarelli et al. 2020).

### Temporal pattern

**Figure 1.**
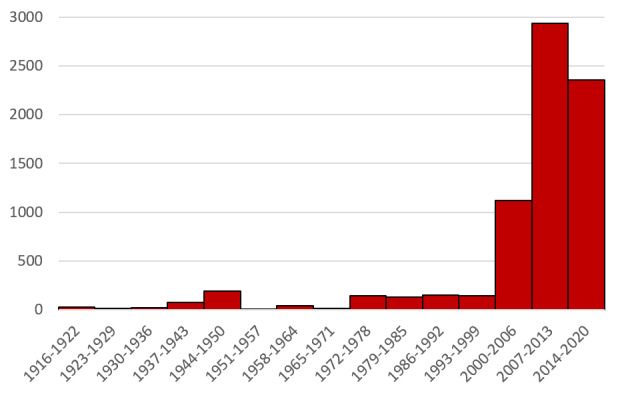
Temporal distribution of Argentinean triatomine occurrence data. Frequency distribution of the number of occurrence data per 7-year intervals. Records with temporal information expressed as a time range that spanned both sides of the pre- and post-2000 time boundary (e.g. 1998-2006) were assigned to the time interval with the largest number of years of collection.

The compiled information for Argentina spans the period from 1918 to 2019, with 70% of the occurrences corresponding to the last 20 years (Fig. 1, Table 1). Considering that most of the records belong to the latter time interval and to describe better the information, the dataset was split into two periods, (a) a pre-2000 period (up to and including 2000) and (b) a post-2000 period (2001 to the present).

There are 2930 occurrence records in the pre-2000 period corresponding to information on 17 triatomine species, compared to 6858 records corresponding to 13 triatomine species in the post-2000 period. The species missing from the post-2000 period are *P. megistus*, *P. rufotuberculatus*, *T. limai* and *T. rubrofasciata* (Table 1). In the pre-2000 period, the largest contribution of occurrence data corresponds to *T. infestans* (43.36%), *T. guasayana* (10.34%), *P. guentheri* (6.38%), *Ps. coreodes* (6.11%) and *T. garciabesi* (4.09%), while, in the post-2000 period, the records of *T. infestans* by themselves represent 82.72% of all occurrences (Table 1).

### Types of data sources reviewed

Most of the records (62.79%) were obtained from DataTri and 37.21% from the other three datasets (AMH-CeNDIE/ANLIS, AMH-CeReVe and GeoVin). The main data sources contributing to the pre-2000 records were public repositories (96.66%), while in the post-2000 period, AMH-CeReVe and data provided by colleagues were the data sources with the greatest contribution (51.82% and 33.68%, respectively) (Table 2).

### Habitat type

According to the type of habitat where collected, 29.96% of the species are found inside human dwellings, 65.02% in the vicinity of human dwellings and 5.01% in natural environments (Table 3). A classification, based on the range of habitat types, includes seven categories (Table 3): (1) *Domestic species*, characterised by adults, nymphs, eggs and exuviae present within the house (i.e. the entire life cycle of the insect occurs within human dwellings); (2) *Domiciliary species*, characterised by having their complete cycle inside human dwellings, but maintaining small populations, i.e. showing recent (and relatively poor) adaptation to houses; these populations may disappear from human domiciles without any control intervention; (3) *Domiciliary intrusive species*, characterised by the presence of adult individuals in human dwellings, probably attracted by light or introduced by passive transport, but without evidence of colonisation (i.e. without the presence of nymphs, eggs or exuviae within the domicile); (4) *Peridomestic species*, similar to the *Domestic species*, but completing their entire life cycle around human dwellings; (5) *Peridomiciliary species*, characterised by species that complete their entire life cycle in the vicinity of human dwellings; (6) *Peridomiciliary intrusive species*, characterised by adult individuals reported around human dwellings, but without evidence of colonisation; and (7) *Sylvatic species*, characterised by having their complete life cycle in natural environments.

*Triatoma infestans* is the only species categorised as Domestic and Peridomestic, while *T. garciabesi*, *T. guasayana* and *T. sordida* are mainly Domiciliary and Peridomiciliary species. In the case of *T. eratyrusiformis*, *T. patagonica* and *T. platensis*, they are mainly Peridomiciliary species, whereas *P. geniculatus* and *P. guentheri* are mainly Peridomiciliary intrusive species. Additionally, even though the relative frequency of records for each species in natural environments are heterogeneous, *Ps. coreodes*, *T. breyeri and T. delpontei* are Sylvatic species, with the maximum percentage of nymphal stages recorded in this habitat type (Table 3).

### Geographic distributions and species richness pattern

The occurrence data for triatomine species are distributed over 15 ecoregions, amongst which those with highest number of species occurrences in both periods are Dry Chaco, Humid Chaco, Espinal, Plains and Plateaus Monte and Hills and Bossoms Monte (Suppl. material 1).

**Figure 2.**
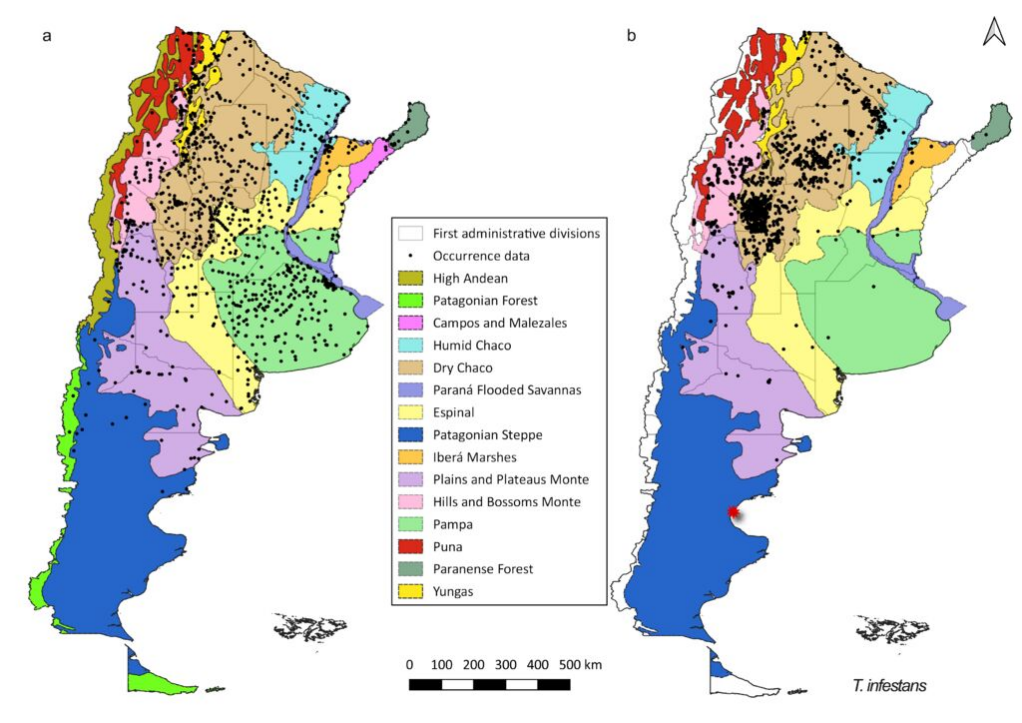
Distribution of occurrence data for *T. infestans*. (a) pre-2000 records (b) post-2000 records. Species occurrence data are shown as black dots. Coloured areas represent the ecoregions. Red star is a questionable occurrence (Piccinali et al. 2010).

The species with widest geographic distribution is *T. infestans* (Fig. 2), with the highest number of records in both periods (Table 1). For the pre-2000 period, records of *T. infestans* are distributed in all continental ecoregions, while in the post-2000 period, there are no records for this species in the ecoregions High Andes, Patagonian Forest, Paraná Flooded Savannahs and Campos and Malezales ecoregions (Fig. 2). Despite this absence in some ecoregions in the post-2000 records and that the geographic distribution of the species is mainly restricted to one ecoregion (Dry Chaco), for this time period, *T. infestans* has four times the number of records of the pre-2000 period (Table 1).

**Figure 3.**
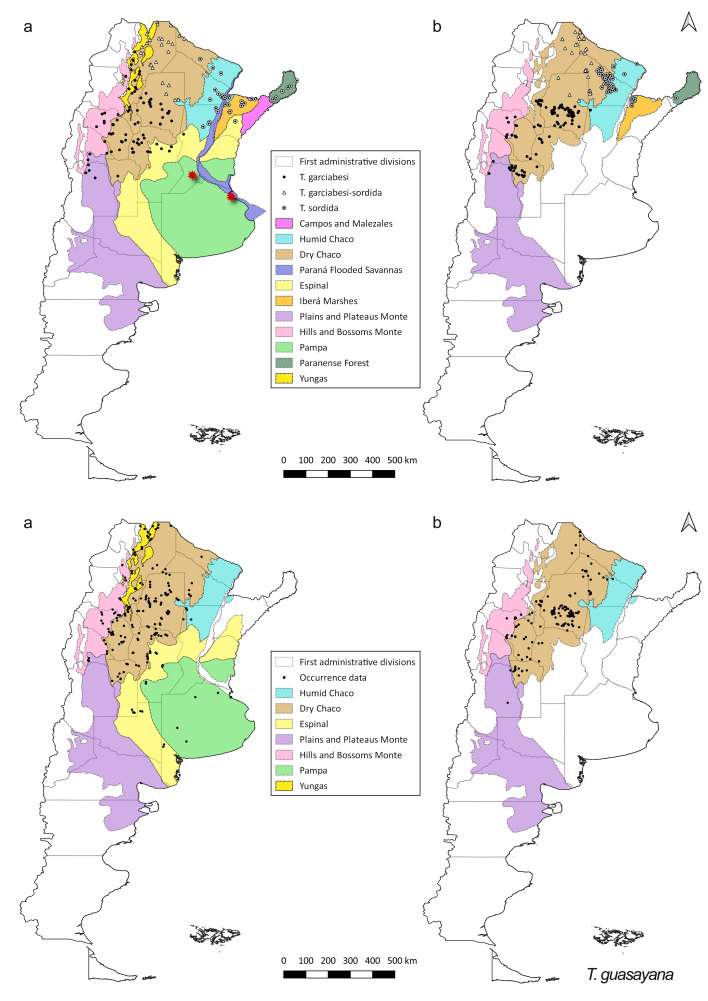
Distribution of occurrence data for *T. garciabesi*, *T. garciabesi*-*T. sordida*, *T. sordida* and *T. guasayana* (a) pre-2000 records (b) post-2000 records. Species occurrence data are shown as black dots (*T. garciabesi* and *T. guasayana*), white circles with black dots (*T. sordida*) and white triangle (*T. garciabesi-T. sordida*). Coloured areas indicate the ecoregions. Red stars are questionable records for *T. sordida* (Abalos and Wygodzinsky 1951, Manso Soto and Martinez 1948).

*Triatoma guasayana* is the species with the second highest number of occurrence data (6.29%), followed by *T. garciabesi* and *T. sordida* (7.65%, including the *T. garciabesi*-*T. sordida* records) (Table 1). The occurrence data for post-2000 for *T. guasayana* records are concentrated in central and north Argentina and mainly restricted to the Dry Chaco ecoregion (Fig. 3). In the case of *T. garciabesi*, the distribution of occurrence data for both periods included north-western Argentina (mainly Dry Chaco), whereas those of *T. sordida* included north-eastern Argentina (mainly Humid Chaco). The geographic distribution of *T. garciabesi* -*T. sordida* is similar for the pre-2000 and post-2000 periods and shows partial overlap between the distributions of both species (Fig. 3).

**Figure 4.**
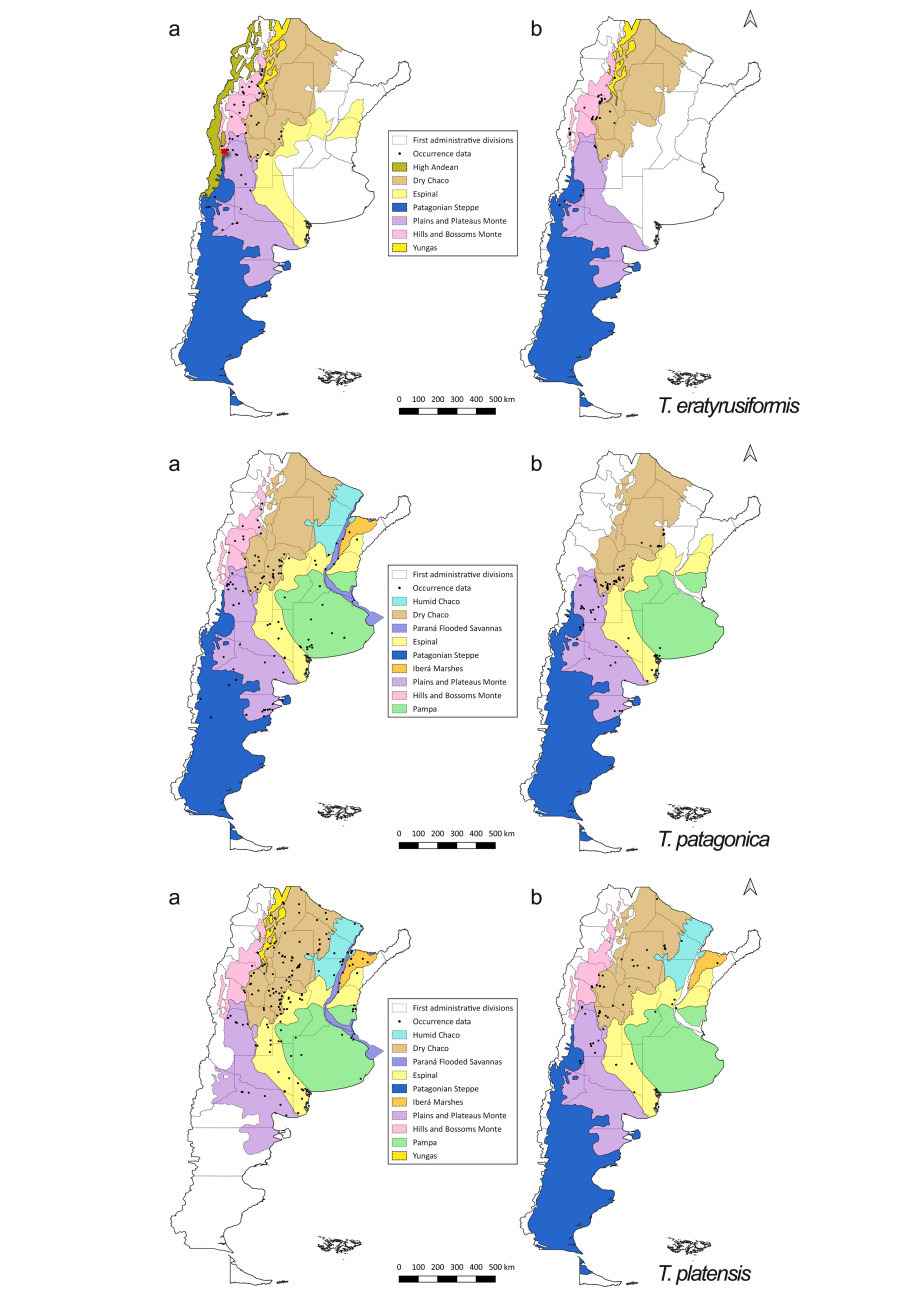
Distribution of occurrence data for *T. eratyrusiformis*, *T. patagonica* and *T. platensis*. (a) pre-2000 records (b) post-2000 records. Species occurrence data are shown as black dots. Coloured areas represent the ecoregions. Red star is a questionable record for *T. eratyrusiformis* (*Bachmann 1999*).

*Triatoma eratyrusiformis* presented similar distribution patterns of occurrence between the pre-2000 and post-2000 periods. For *T. patagonica*, occurrences in the post-2000 period were restricted to the southern Dry Chaco, Plains and Plateaus Monte and the Espinal ecoregion in southern Buenos Aires Province (Fig. 4). Similarly, occurrence data for *T. platensis* for the post-2000 period were restricted mainly to the ecoregions Dry Chaco and Plains and Plateaus Monte (Fig. 4).

**Figure 5.**
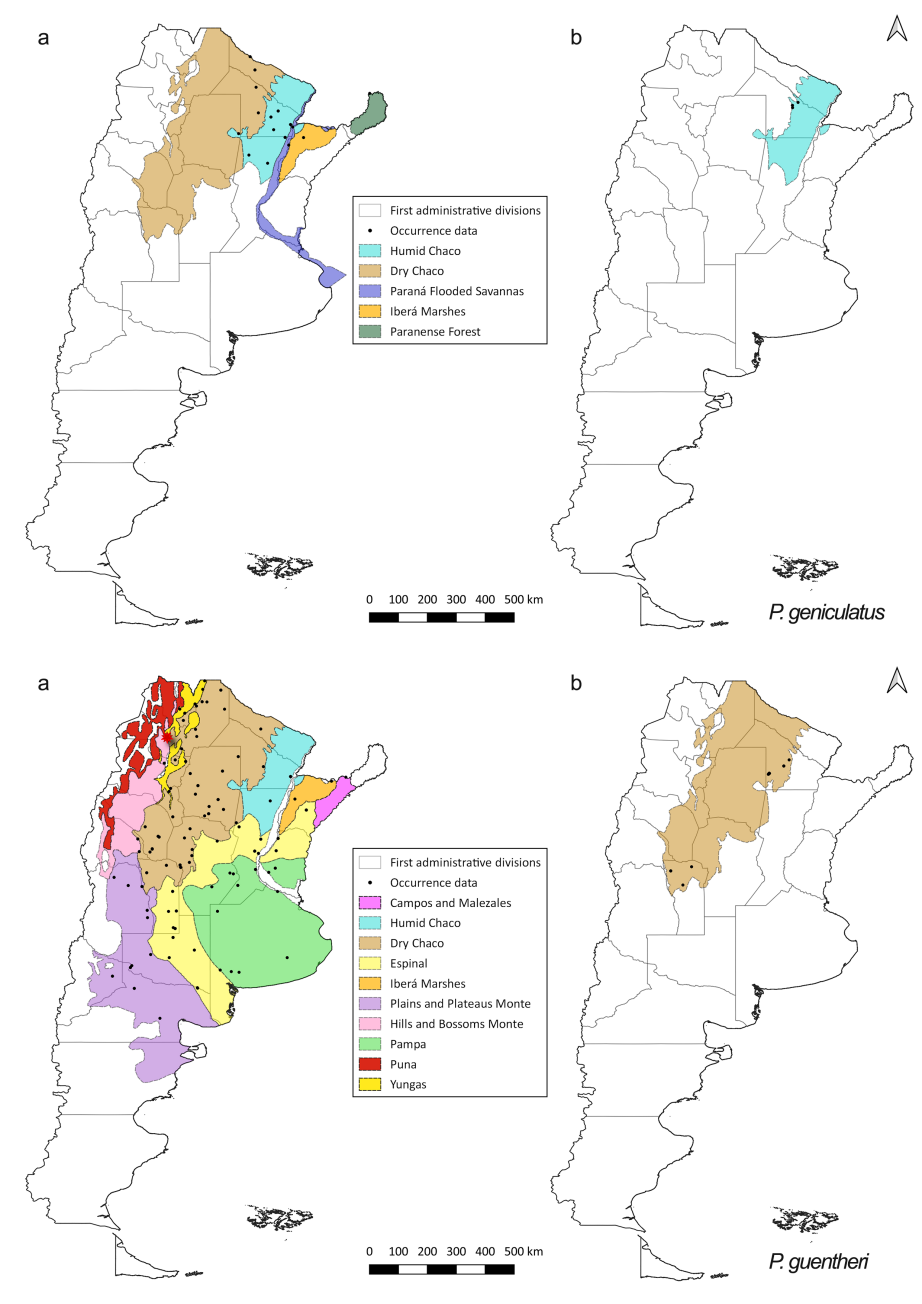
Distribution of occurrence data for *P. geniculatus* and *P. guentheri* (a) pre-2000 records (b) post-2000 records. Species occurrence data are shown as black dots. Coloured areas represent the ecoregions. Red star is a questionable record for *P. guentheri* (Carcavallo et al. 1994).

There are fewer records of *P. geniculatus* and *P. guentheri* in the post-2000 than in the pre-2000 period, with occurrences restricted to the Humid and Dry Chaco ecoregions, respectively (Fig. 5). Meanwhile, *P. guentheri* is the species that shows greatest reduction of its distribution between the pre-2000 and the post-2000 periods (Fig. 5).

**Figure 6.**
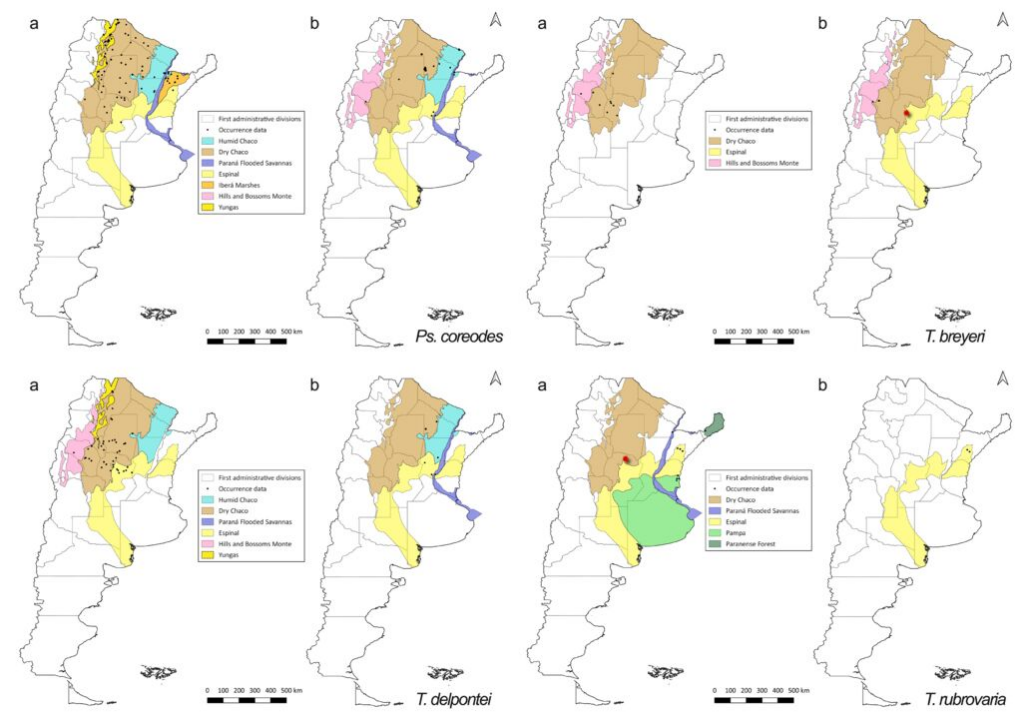
Distribution of occurrence data for *Ps. coreodes*, *T. breyeri*, *T. delpontei* and *T. rubrovaria* (a) pre-2000 records (b) post-2000 records. Species occurrence data are shown as black dots. Coloured areas represent the ecoregions. Red stars are questionable records for *T. breyeri* (Moreno et al. 2006) and for *T. rubrovaria* (*Carcavallo and Martinez 1968*).

The species *Ps. coreodes*, *T. breyeri*, *T. delpontei* and *T. rubrovaria* show reduction of their geographic ranges from pre-2000 to post-2000 periods, with scarce post-2000 records (Fig. 6).

**Figure 7.**
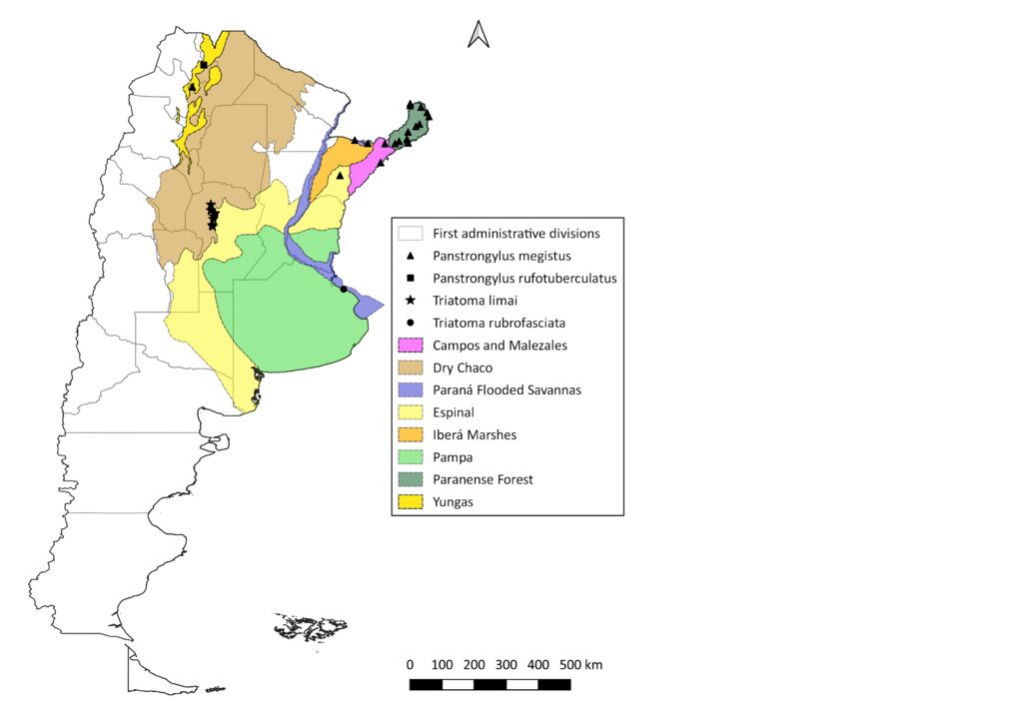
Distribution of occurrence data for triatomine species with only pre-2000 information available. Species occurrence data are shown as black triangles (*P. megistus*), black squares (*P. rufotuberculatus*), black stars (*T. limai*) and black dots (*T. rubrofasciata*). Coloured areas represent the ecoregions.

No reports of occurrence were found for *P. megistus*, *P. rufotuberculatus*, *T. limai* and *T. rubrofasciata* in the post-2000 period; thus, Fig. 7 shows only the geographic information corresponding to the pre-2000 period for these species. *Panstrongylus megistus* has 27 records; *P. rufotuberculatus*, one record; *T. limai*, nine records; and *T. rubrofasciata*, one record (Table 1).

**Figure 8.**
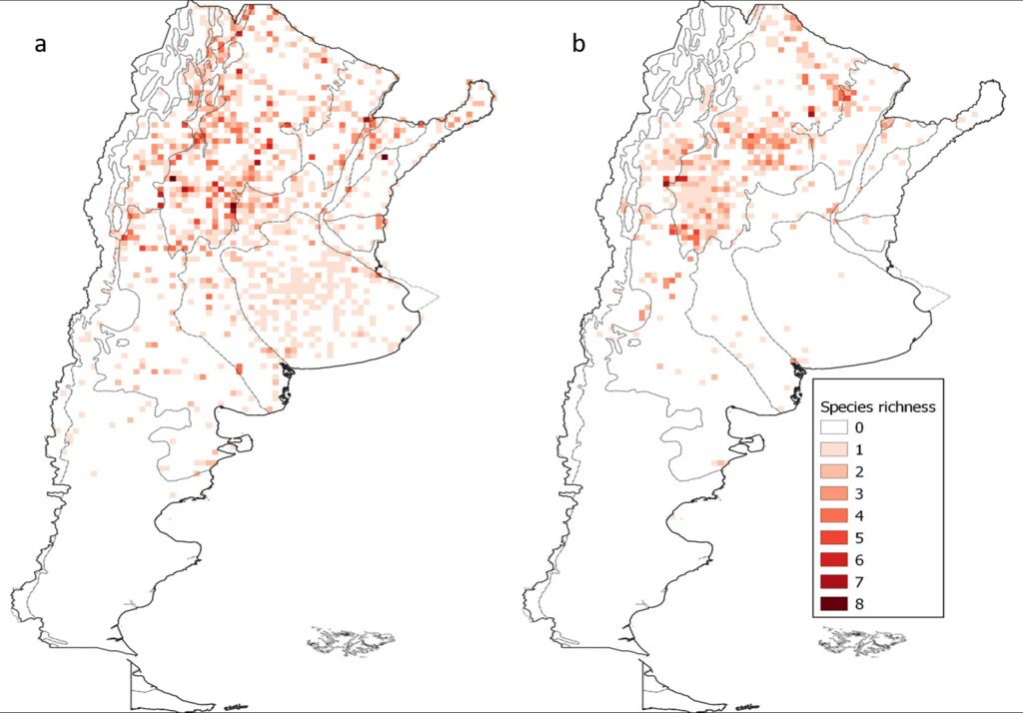
Spatial pattern of Argentinean triatomine species richness. (a) pre-2000 occurrence data and (b) post-2000 occurrence data. The colour gradient corresponds to the number of species present in each cell. Dashed lines indicate limits between ecoregions.

These data show a trend of reduction in species richness of triatomines over the last 20 years in central-western Argentina (an area corresponding mainly to the southern and central Dry Chaco ecoregion) (Fig. 8). The maximum number of species sharing a cell in the pre-2000 period is eight species, while this maximum decreased to seven in the post-2000 period. Furthermore, for the pre-2000 period, high species richness values were found in several ecoregions of Argentina, whereas in the post-2000 period, they occurred mainly in only four areas within the Dry Chaco (Fig. 8b). The species with greatest contribution to overall richness in both periods and in decreasing order are *T. infestans*, *T. platensis*, *T. guasayana* and *T. garciabesi* (details in Table 1).

## Temporal coverage

**Formation period:** 1912-2019.

## Usage licence

### Usage licence

Creative Commons Public Domain Waiver (CC-Zero)

## Data resources

### Data package title

Datos de ocurrencia de triatominos presentes en Argentina del Laboratorio de Triatominos del CEPAVE (CONICET-UNLP)

### Resource link

https://ipt.mincyt.gob.ar/resource?r=triatominosargentinos1&v=1.2

### Alternative identifiers

https://www.gbif.org/dataset/94adad5f-fb11-426f-93e0-3dd02f3ccd1d

### Number of data sets

1

### Data set 1.

#### Data set name

Datos de ocurrencia de triatominos presentes en Argentina del Laboratorio de Triatominos del CEPAVE (CONICET-UNLP)

#### Data format

Darwin Core Archive

#### Number of columns

45

#### Download URL

https://doi.org/10.15468/ak8aax

#### 

**Data set 1.** 

Column label	Column description
occurrenceID	The globally unique identifier for the occurrence.
dcterms:type	The nature of the resource.
dcterms:modified	The most recent date-time on which the resource was changed.
dcterms:language	A language of the resource.
institutionCode	The name of the institution having custody of the resource.
collectionCode	The name identifying the dataset from which the record was derived.
basisOfRecord	The specific nature of the data record.
catalogNumber	An unique identifier for the record within the dataset.
higherClassification	A concatenated list of taxa names terminating at the rank immediately superior to the taxon referenced in the taxon record.
kingdom	The full scientific name of the kingdom in which the taxon is classified.
phylum	The full scientific name of the phylum in which the taxon is classified.
class	The full scientific name of the class in which the taxon is classified.
order	The full scientific name of the order in which the taxon is classified.
family	The full scientific name of the family in which the taxon is classified.
genus	The full scientific name of the genus in which the taxon is classified.
specificEpithet	The name of the species epithet of the scientificName.
scientificNameAuthorship	The authorship information for the scientificName formatted according to the conventions of the applicable nomenclatural Code.
scientificName	The full scientific name.
taxonRank	The taxonomic rank of the most specific name in the scientificName.
recordedBy	A person, group or organisation responsible for recording the original Occurrence.
individualCount	The number of individuals present at the time of the Occurrence.
sex	The sex of the biological individual(s) represented in the Occurrence.
lifeStage	The life stage of the biological individual(s) at the time the Occurrence was recorded.
year	The four-digit year in which the Event occurred.
month	The ordinal month in which the Event occurred.
day	The integer day of the month on which the Event occurred.
habitat	A category of the habitat in which the Event occurred.
samplingProtocol	The name of, reference to, or description of the method or protocol used during an Event.
samplingEffort	The amount of effort expended during an Event.
higherGeography	A concatenated list of geographic names less specific than the information captured in the locality term.
continent	The name of the continent in which the Location occurs.
country	The name of the country in which the Location occurs.
countryCode	The standard code for the country in which the Location occurs.
stateProvince	The name of the next smaller administrative region than country in which the Location occurs.
municipality	The full, unabbreviated name of the next smaller administrative region than county in which the Location occurs.
locality	The specific description of the place.
verbatimLocality	The original textual description of the place.
decimalLatitude	The geographic latitude (in decimal degrees) of the geographic centre of a Location.
decimalLongitude	The geographic longitude (in decimal degrees) of the geographic centre of a Location.
geodeticDatum	The spatial reference system (SRS) upon which the geographic coordinates given in decimalLatitude and decimalLongitude are based.
georeferencedBy	Names of people, groups or organisations who determined the georeference for the Location.
georeferenceSource	A list of resources used to georeference the Location.
associatedReferences	Identifier (publication, bibliographic reference, global unique identifier, URI) of literature associated with the Occurrence.
coordinateUncertaintyInMetres	The horizontal distance (in metres) from the given decimalLatitude and decimalLongitude describing the smallest circle containing the whole of the Location.
eventDate	The date-time or interval when the event was recorded.

## Additional information

### Conclusions and prospects

We compiled a large amount of occurrence data and associated information and integrated it into a single database to better understand the geographic distribution of triatomine species present in Argentina over the last 100 years, as well as the temporal variation of these distributions.

One of our key findings was that the number of records in the post-2000 period almost tripled the number of those from the pre-2000 period. While for the pre-2000 period, public repositories (mainly records from researcher’s fieldwork published in scientific journals) were the major data sources providing spatial information, in the last two decades, the balance shifted towards records provided by the CeReVe, as well as focused field campaigns carried out by several research groups, which were the major data sources for the post-2000 period. However, these sources have become increasingly focused on the species with major epidemiological importance, mainly *T. infestans* and few records provide information about other species. We propose the following hypotheses that may account for this difference: (a) the results of chemical control and vector eradication campaigns that were carried out through the creation of the INCOSUR initiative in 1992 (http://www.paho.org) became evident only in the post-2000 period; (b) in the 1997-2007 period, Argentinean researchers received the highest number of grants from WHO Special Programme for Research and Training in Tropical Diseases (TDR) and the Pan American Health Organization (PAHO), which resulted also in the highest number of related publications in peer-reviewed scientific journals (Carbajal de la Fuente and Yadón 2013); and (c) in the last 20 years, the CeReVe began to play a major role as one of the most important institutions for the compilation of triatomine records of Argentina, becoming the main source of records for this country in the post-2000 period.

Another of the key findings concerns the number of triatomine species present in Argentina. According to Carcavallo et al. (1998) and Ceccarelli et al. (2018), there are 17 triatomine species mentioned for Argentina. However, in this analysis, we found that this number of species is valid only for the pre-2000 period, given that, for at least in the last 20 years, there are no records for *P. megistus*, *P. rufotuberculatus*, *T. limai* or *T. rubrofasciata*. In the case of *P. megistus*, the last record found for Argentina corresponds to 1995 (Damborsky et al. 2001); however, the records mentioned for Brazil (Ceccarelli et al. 2018) and Bolivia (Rojas-Cortez 2007) in the post-2000 period, suggest that this species might still be present in Argentina. Although only one record was found for *P. rufotuberculatus* in the Yungas ecoregion (Salomón et al. 1999) for the pre-2000 period, the species has been detected in Tarija (Bolivia) several times (Rojas-Cortez 2007), leading us to consider that the scarcity of data may be due to a lack of sampling in that ecoregion. Similarly, *T. limai* was mentioned in only two publications for Argentina (Abalos and Wygodzinsky 1951, Carcavallo and Martinez 1968), while Del Ponte (1929) mentioned that it is distributed in Brazil, but without specifying a locality. Finally, given that *T. rubrofasciata* is a cosmopolitan species suspected of having been transported on ships to some countries, we concluded that it is possible that this species arrived accidentally to Argentina at some point. The only record of *T. rubrofasciata* is considered as doubtful because Neiva (1914), Larrousse (1924) and Del Ponte (1930) do not agree on the location of the record. Abalos and Wygodzinsky (1951) do not mention *T. rubrofasciata* in Argentina and Carcavallo et al. (1965) proposed to remove it from the list of Argentinean triatomines, since apparently it was found occasionally and was never able to colonise any environment in Argentina. Therefore, we consider that the specimens of *T. limai*, allegedly found in Argentina, do not belong to the species described by del Ponte in 1930 and as there has been no record of *T. rubrofasciata* in Argentina for more than 100 years, we chose to remove both species from the current list of Argentinean triatomines. On the other hand, we decided to keep *P. megistus* and *P. rufotuberculatus*, judging that the small number of occurrences in the last 20 years can be attributed to poor sampling.

In the case of insect vectors, it is common for those species with greater public health importance (e.g. domiciliated species) to receive more attention, as in the case of *T. infestans* that makes up 70.98% of all the occurrence data and has the largest geographic distribution in Argentina. The occurrence records for some species are also biased towards certain regions and habitats: occurrence data of most species show distributions restricted mainly to the Dry and Humid Chaco ecoregions in the post-2000 period, with *P. guentheri* showing the greatest reduction of occurrence distribution, from 10 ecoregions in the pre-2000 period to only one in the post-2000 period. Undoubtedly, this phenomenon involves not only the usual tendency of researchers to survey specific or more accessible places (Sastre and Lobo 2009), but also the fact that some areas receive more attention from vector control programmes (Carbajal de la Fuente and Yadón 2013). Thus, this uneven distribution of missing data, influenced by various factors, results in a less accurate representation of the actual occurrence patterns. Thus, the known distribution of a species provides a view of its distribution that is at best incomplete, if not actually misleading. A critical step towards improving this picture is to reinforce the efforts in unsampled and under-sampled areas, considering not only the domiciliary and peridomiciliary species, but also the sylvatic ones. When compiling the distribution data from public sources, we often found it useful to allocate attention to species and regions depending on data availability, because databasing sylvatic species from an under-sampled region provides more novel information than duplicating records for well-known species (domiciliary species) and regions (endemic areas). Applications based on citizen-science projects, such as GeoVin, aim to promote an increase in reports of sylvatic species, as well as an update of records of species more commonly known for their major vector importance (such as *T. infestans*) from locations outside those areas where reports are commonly made. We also found that unpublished data, such as the records provided by CeReVe and CeNDIE/ANLIS, from colleagues or from studies that are not focused on geography (e.g. molecular, morphometric etc.) are highly-valuable sources of geographic records and we recommend and encourage them to publish their data with geographic coordinates. We also advise them to publish those data in public databases (instead of as part of scientific articles only) because online databases of observations from field studies not only facilitate data gathering, but also contribute to guarantee the persistence and availability of those data.

Finally, on the basis of the information about habitat and the categorisation presented here, we show that, although the greatest proportion of records in all habitat types corresponds to *T. infestans*, there are also other species, namely *T. sordida*, *T. garciabesi* and *T. guasayana*, that occur as both adults and nymphs inside and around human dwellings. In the case of *T. eratyrusiformis, T. patagonica* and *T. platensis*, the records of occurrence within human dwellings include adult individuals only, but these species also occur as nymphs in the vicinity of human dwellings. The categorisation used in this work could contribute to the development of vector risk indices and, combined with the maps of species distributions, could be extremely useful for decision-makers in health organisations, since the risk of these insects acting as vectors of *T. cruzi* to result in Chagas disease is not only due to the presence of the triatomines themselves, but also depends on their proximity to and colonisation of human dwellings.

In summary, we used an approach, based on occurrence data analysis, to assess temporal, spatial and ecological patterns of the triatomines present in Argentina. Our results provide updated information regarding various aspects of Argentinean triatomines that will improve the ways in which these species are identified, controlled and managed in Argentina. Two major outcomes of this work are, on one hand, the inclusion of 15 species in the updated list of Argentinean triatomines: *P. geniculatus*, *P. guentheri*, *P. megistus*, *P. rufotuberculatus*, *Ps. coreodes*, *Triatoma breyeri*, *T. delpontei*, *T. eratyrusiformis*, *T. garciabesi*, *T. guasayana*, *T. infestans*, *T. patagonica*, *T. platensis*, *T. rubrovaria* and *T. sordida*. On the other hand, the database analysed will add records to those of DataTri, thus becoming the largest open access database of American triatomines with around 30000 records so far, which not only represents a fundamental resource for Chagas disease decision-making, but also contributes to the initiatives related to open access data. Ongoing investment in national records for vector control is crucial for the continuous growth of the datasets upon which our analyses are based. The methods that we developed and implemented can be easily transferred to and applied in other countries where these types of data are available, thus improving our understanding of existing triatomine information.

## Supplementary Material

72FCBE2D-B118-52A9-B075-E39BF94DE123 10.3897/BDJ.8.e58076.suppl1 Supplementary material 1 Presence of each triatomine species in the Argentinean ecoregions. Data type Occurrences File: oo_465776.html https://binary.pensoft.net/file/465776 Soledad Ceccarelli, Agustín Balsalobre, María Eugenia Cano, Delmi Canale, Patricia Lobbia, Raúl Stariolo, Jorge Eduardo Rabinovich & Gerardo Aníbal Marti

## Figures and Tables

**Table 1.** Temporal information for each Argentinean triatomine species. Number of records (excluding those in areas outside the Argentinean borders) of pre-2000, post-2000 and totals occurrences with their respective percentages and information interval for both periods.

**Triatomine species**	**Pre-2000** **information** **interval**	**N° of pre-2000 records**	% **of pre-2000 records**	**Post-2000** **information** **interval**	**N° of post-2000 records**	% **of post-2000 records**	**N° of total records**	% **of total records**
*P. geniculatus*	1945-1995	21	0.72	2008-2015	4	0.06	25	0.26
*P. guentheri*	1927-1994	187	6.38	2006-2018	27	0.39	214	2.19
*P. megistus*	1945-1995	27	0.92	-	-	-	27	0.28
*P. rufotuberculatus*	1997	1	0.03	-	-	-	1	0.01
*Ps. coreodes*	1934-1999	179	6.11	2006-2017	66	0.96	245	2.50
*T. breyeri*	1940-1989	12	0.41	2008-2010	14	0.20	26	0.27
*T. delpontei*	1943-1981	71	2.42	2006-2015	24	0.35	95	0.97
*T. eratyrusiformis*	1929-1999	85	2.90	2006-2019	50	0.73	135	1.38
*T. garciabesi*	1929-1999	120	4.09	2003-2018	219	3.18	339	3.46
*T. garciabesi-T. sordida*	1936-1998	77	2.62	2002-2017	72	1.05	149	1.52
*T. guasayana*	1936-2000	303	10.34	2001-2018	313	4.56	616	6.29
*T. infestans*	1919-2000	1273	43.36	2001-2019	5673	82.72	6946	70.96
*T. limai*	1918-1968	9	0.31	-	-	-	9	0.09
*T. patagonica*	1927-1998	176	6.01	2003-2019	109	1.59	285	2.91
*T. platensis*	1936-1999	292	9.97	2003-2018	96	1.40	388	3.96
*T. rubrofasciata*	1924	1	0.03	-	-	-	1	0.01
*T. rubrovaria*	1942-1999	24	0.82	2001-2003	2	0.03	26	0.27
*T. sordida*	1935-1999	72	2.46	2003-2018	189	2.75	261	2.67
**Total**	**1918-2000**	**2930**		**2001-2019**	**6858**		**9788**	

**Table 2.** Types of data sources reviewed. The chart presents the number of records compiled from each information source type and the percentage of contribution from each information source in each period (pre- and post-2000). The “New data sets” rows correspond to sources of information obtained after the publication of DataTri.

	**Data source type**	**Number of pre-2000 records**	% **of pre-2000 data contribution**	**Number of post-2000 records**	% **of post-2000 data contribution**	**Total number of records**	% **of total data contribution**
**New data sets**	AMH-CeNDIE/ANLIS	0	0	24	0.35	24	0.25
	AMH-CeReVe	0	0	3554	51.82	3554	36.31
	Citizen science - GeoVin	0	0	64	0.93	64	0.65
		**0**	**0**	**3642**	**53.11**	**3642**	**37.21**
**DataTri**	Personal field work - CEPAVE	0	0	395	5.76	395	4.04
	Data provided by colleagues	98	3.34	2310	33.68	2408	24.60
	Public repositories	2832	96.66	511	7.45	3343	34.15
		**2930**	**100**	**3216**	**46.89**	**6146**	**62.79**
	Total	**2930**		**6858**		**9788**	

**Table 3.** Classification of triatomine species in the Argentinean territory according to the level of domiciliation/intrusion in human dwellings and natural environment. The percentage of records in each habitat type represents the number of records for each species relative to the total records for each habitat type. The percentage of records with presence of nymphal stages represents the number of records of each species with nymphal stages presence relative to the total records for each species in each species category.

**Habitat type** **(number of records)**	**Species c ategories**	**Triatomine species**	**Percentage of records in each habitat type**	**Percentage of records with presence of nymphal stage**
Inside human dwellings (Domicile) (N = 2193)	Domestic species	*T. infestans*	93.75 (N = 2056)	36.72 (N = 755)
	Domiciliary species	*T. sordida*	1.96 (N = 43)	55.81 (N = 24)
		*T. guasayana*	1.65 (N = 36)	27.78 (N = 10)
		*T. garciabesi*	1.28 (N = 28)	57.14 (N = 16)
	Domiciliary intrusive species	*T. eratyrusiformis*	0.27 (N = 6)	NA
		*T. patagonica*	0.5 (N = 11)	NA
		*P. guentheri*	0.37 (N = 8)	NA
		*T. platensis*	0.18 (N = 4)	NA
		*P. geniculatus*	0.05 (N = 1)	NA
Around human dwellings (Peridomicile) (N = 4759)	Peridomestic species	*T. infestans*	84.28 (N = 4011)	36.60 (N = 1468)
	Peridomiciliary species	*T. garciabesi*	4.58 (N = 218)	85.78 (N = 187)
		*T. guasayana*	4.22 (N = 201)	78.11 (N = 157)
		*T. sordida*	4.24 (N = 202)	68.32 (N = 138)
		*T. patagonica*	1.72 (N= 82)	62.2 (N = 51)
		*T. eratyrusiformis*	0.29 (N = 14)	50 (N = 7)
		*T. platensis*	0.5 (N = 24)	45.83 (N = 11)
	Peridomiciliary intrusive species	*P. guentheri*	0.06 (N = 3)	NA
		*P. geniculatus*	0.04 (N = 2)	NA
Natural environment (N = 367)	Sylvatic species	*T. infestans*	18.53 (N = 68)	14.71 (N = 10)
		*Ps. coreodes*	17.17 (N = 63)	42.86 (N = 27)
		*T. platensis*	16.35 (N = 60)	50 (N = 30)
		*T. guasayana*	16.08 (N = 59)	30.51 (N = 18)
		*T. garciabesi*	8.99 (N = 33)	24.24 (N = 8)
		*T. delpontei*	5.99 (N = 22)	40.91 (N = 9)
		*T. sordida*	5.18 (N = 19)	26.32 (N = 5)
		*P. guentheri*	3.81 (N = 14)	NA
		*T. breyeri*	3.81 (N = 14)	64.29 (N = 9)
		*T. eratyrusiformis*	2.72 (N = 10)	NA
		*T. patagonica*	0.54 (N = 2)	NA
		*T. rubrovaria*	0.54 (N = 2)	50 (N = 1)
		*P. geniculatus*	0.27 (N = 1)	100 (N = 1)

## References

[B5908887] Abalos J. W., Wygodzinsky P. (1951). Las Triatominae Argentinas. Instituto de Medicina Regional. Universidad Nacional de Tucumán.

[B5912922] Bachmann Axel (1999). Catálogo de los tipos de Heteroptera (Insecta) conservados en el Museo Argentino de Ciencias Naturales. Revista del Museo Argentino de Ciencias Naturales, nueva serie.

[B5912618] Bérenger Jean-Michel, Pluot-Sigwalt Dominique, Pagès Frédéric, Blanchet Denis, Aznar Christine (2009). The triatominae species of French Guiana (Heteroptera: Reduviidae). Memórias do Instituto Oswaldo Cruz.

[B5913069] Bivand R., Lewin-Koh N. (2018). maptools: Tools for Reading and Handling Spatial Objects.. http://cran.r-project.org/package=maptools.

[B5912765] Boakes Elizabeth H., McGowan Philip J. K., Fuller Richard A., Chang-qing Ding, Clark Natalie E., O'Connor Kim, Mace Georgina M. (2010). Distorted views of biodiversity: Spatial and temporal bias in species occurrence data. PLoS Biology.

[B5913021] Burkart R., Bárbaro N. O., Sánchez R. O., Gómez D. A. (1999). Eco-regiones de la Argentina. https://sib.gob.ar/archivos/Eco-Regiones_de_la_Argentina.pdf.

[B5912950] Carbajal de la Fuente Ana Laura, Yadón Zaida E. (2013). A scientometric evaluation of the Chagas disease implementation research programme of the PAHO and TDR. PLoS Neglected Tropical Diseases.

[B5925019] Carcavallo R. U., Cichero J. A., Martínez A., Prosen A. F., Ronderos R. (1965). Una nueva especie del género *Triatoma* Laporte (Hemiptera, Reduviidae, Triatominae).

[B5912985] Carcavallo R. U., Martinez A. (1968). Enfermedad de Chagas y sus transmisores. Comunicaciones Científicas. Junta de Investigaciones Científicas de las Fuerzas Armadas Argentinas..

[B5912931] Carcavallo R. U., Martinez A., Canale D., Mena Segura C. A., Casas S. I. (1994). Notas sobre la biologia, ecologia y distribución geográfica del *Panstrongylus
guentheri* Berg, 1879. Entomología y Vectores.

[B5908913] Carcavallo R. U., Curto de Casas S. I., Sherlock I. A., Galíndez Girón I., Jurberg J., Galvao C., Mena Segura C. A., Noireau F., Carcavallo R. U., Galíndez Girón I., Jurberg J., Lent H. (1998). Geographical distribution and alti-latitudinal dispersion of Triatominae. Atlas of Chagas' disease vectors in the Americas.

[B5912714] Cazorla-Perfetti D. J., Nieves-Blanco E. E. (2010). Triatominos de Venezuela: aspectos taxonómicos, biológicos, distribución geográfica e importancia médica. Avances Cardiológicos.

[B5912882] Ceccarelli Soledad, Balsalobre Agustín, Medone Paula, Cano María Eugenia, Gurgel Gonçalves Rodrigo, Feliciangeli Dora, Vezzani Darío, Wisnivesky-Colli Cristina, Gorla David E, Marti Gerardo A, Rabinovich Jorge E (2018). DataTri, a database of American triatomine species occurrence. Scientific Data.

[B5928480] Ceccarelli S, Balsalobre A, Cano M E, Vicente M E, Rabinovich J E, Medone P, Cochero J, Canale D, Lobbia P, Stariolo R, Marti G A (2020). Datos de ocurrencia de triatominos presentes en Argentina del Laboratorio de Triatominos del CEPAVE (CONICET-UNLP).

[B6299670] Chapman Arthur, Wieczorek John (2006). Guide to Best Practices for Georeferencing. GBIF Secretariat.

[B5912669] Chávez J. (2006). Contribución al estudio de los triatominos del Perú: Distribución geográfica, nomenclatura y notas taxonómicas. Anales de la Facultad de Medicina. Universidad Nacional Mayor de San Marcos.

[B5912959] Damborsky M. P., Bar M. E., Oscherov E. B. (2001). Detección de triatominos (Hemiptera: Reduviidae) en ambientes domésticos y extradomésticos. Cadernos de Saúde Pública.

[B5912994] Del Ponte E. (1929). Algunas especies nuevas del género *Triatoma*. Boletin de la Sociedad Entomológica Argentina.

[B5924980] Del Ponte E. (1930). Catálogo descriptivo de los géneros *Triatoma* Lap., *Rhodnius* Stål, e *Eratyrus* Stål. Revista del Instituto Bacteriológico del Departamento Nacional de Higiene.

[B5912569] Galvão C. (2014). Vetores da Doença de Chagas no Brasil.

[B5908878] Galvão Cleber, Justi Silvia A. (2015). An overview on the ecology of Triatominae (Hemiptera: Reduviidae). Acta Tropica.

[B5912609] Guhl Felipe, Aguilera Germán, Pinto Néstor, Vergara Daniela (2007). Actualización de la distribución geográfica y ecoepidemiología de la fauna de triatominos (Reduviidae: Triatominae) en Colombia. Biomédica.

[B5913037] Gurgel-Gonçalves R., Ferreira J. B.C., Rosa A. F., Bar M. E., Galvão C. (2010). Geometric morphometrics and ecological niche modelling for delimitation of near-sibling triatomine species. Medical and Veterinary Entomology.

[B5912866] Hay Simon I., Battle Katherine E., Pigott David M., Smith David L., Moyes Catherine L., Bhatt Samir, Brownstein John S., Collier Nigel, Myers Monica F., George Dylan B., Gething Peter W. (2013). Global mapping of infectious disease. Philosophical Transactions of the Royal Society B: Biological Sciences.

[B5912630] Hiwat Hélène (2014). Triatominae species of Suriname (Heteroptera: Reduviidae) and their role as vectors of Chagas disease. Memórias do Instituto Oswaldo Cruz.

[B5912732] Hortal Joaquín, de Bello Francesco, Diniz-Filho José Alexandre F., Lewinsohn Thomas M., Lobo Jorge M., Ladle Richard J. (2015). Seven shortfalls that beset large-scale knowledge of biodiversity. Annual Review of Ecology, Evolution, and Systematics.

[B5912723] Justi Silvia A., Galvão Cleber (2017). The evolutionary origin of diversity in Chagas disease vectors. Trends in Parasitology.

[B5912823] Kraemer Moritz U. G., Sinka Marianne E., Duda Kirsten A., Mylne Adrian, Shearer Freya M., Brady Oliver J., Messina Jane P., Barker Christopher M., Moore Chester G., Carvalho Roberta G., Coelho Giovanini E., Van Bortel Wim, Hendrickx Guy, Schaffner Francis, Wint G. R. William, Elyazar Iqbal R. F., Teng Hwa-Jen, Hay Simon I. (2015). The global compendium of Aedes aegypti and Ae. albopictus occurrence. Scientific Data.

[B5925010] Larrousse F. (1924). Triatomes díAsie: description díune nouvelle espèce *Triatoma
bouvieri* n.sp.. Annales de Parasitologie Humaine et Comparé.

[B5912752] Lomolino Mark V., Lomolino M. V., Heaney L. R. (2004). Conservation biogeography. Frontiers of Biogeography: New directions in the geography of nature.

[B5912913] Manso Soto A. E., Martinez A. (1948). La colección de Triatómidos de la M.E.P.R.A.. Misión de Estudios de Patología Regional Argentina.

[B5908905] Martinez A, Cichero J. A. (1972). Los Vectores de la Enfermedad de Chagas. Lucha contra los mismos en la Argentina.

[B5912941] Moreno Mariana Laura, Gorla David, Catalá Silvia (2006). Association between antennal phenotype, wing polymorphism and sex in the genus *Mepraia* (Reduviidae: Triatominae). Infection, Genetics and Evolution.

[B5924972] Neiva A (1914). Revisão do gênero *Triatoma* Lap.

[B5913047] Panzera Francisco, Pita Sebastián, Nattero Julieta, Panzera Yanina, Galvão Cleber, Chavez Tamara, Rojas De Arias Antonieta, Cardozo Téllez Lourdes, Noireau François (2015). Cryptic speciation in the *Triatoma
sordida* subcomplex (Hemiptera, Reduviidae) revealed by chromosomal markers. Parasites & Vectors.

[B5912898] Piccinali Romina V., Canale Delmi M., Sandoval Alejandra E., Cardinal Marta V., Jensen Oscar, Kitron Uriel, Gürtler Ricardo E. (2010). *Triatoma
infestans* bugs in Southern Patagonia, Argentina.. Emerging Infectious Diseases.

[B5912846] Pigott David M, Golding Nick, Messina Jane P, Battle Katherine E, Duda Kirsten A, Balard Yves, Bastien Patrick, Pratlong Francine, Brownstein John S, Freifeld Clark C, Mekaru Sumiko R, Madoff Lawrence C, George Dylan B, Myers Monica F, Hay Simon I (2014). Global database of leishmaniasis occurrence locations, 1960–2012. Scientific Data.

[B5913029] Team QGIS Development (2018). QGIS Geographic Information System. http://qgis.osgeo.org.

[B5912595] Ramsey Janine M, Peterson A Townsend, Carmona-Castro Oscar, Moo-Llanes David A, Nakazawa Yoshinori, Butrick Morgan, Tun-Ku Ezequiel, de la Cruz-Félix Keynes, Ibarra-Cerdeña Carlos N (2015). Atlas of Mexican Triatominae (Reduviidae: Hemiptera) and vector transmission of Chagas disease. Memórias do Instituto Oswaldo Cruz.

[B5913061] Team R Core (2019). R: a language and environment for statistical computing. https://www.r-project.org/.

[B5912809] Riddle Brett, Ladle Richard J., Lourie Sara A., Whittaker Robert J., Ladle Richard J., Whittaker Robert J. (2011). Basic Biogeography: Estimating biodiversity and mapping nature. Conservation Biogeography.

[B5912968] Rojas-Cortez Mirko (2007). Triatominos de Bolivia y la enfermedad de Chagas..

[B5912577] Salazar-Schettino P., Rojas-Wastavino G. E., Cabrera-Bravo M., Bucio-Torres M. I., Martínez-Ibarra J. A., Monroy-Escobar M. C., Rodas-Retana A., Guevara-Gómez Y., Vences-Blanco M. O., Ruiz-Hernández A. L., Torres-Gutiérrez E. (2010). Revisión de 13 especies de la familia Triatominae (Hemiptera: Reduviidae) vectores de la enfermedad de Chagas, en México. Journal of the Selva Andina Research Society.

[B5912976] Salomón Oscar D, Ripoll Carlos M, Rivetti Eduardo, Carcavallo Rodolfo U (1999). Presence of *Panstrongylus
rufotuberculatus* (Champion, 1899) (Hemiptera: Reduviidae: Triatominae) in Argentina. Memórias do Instituto Oswaldo Cruz.

[B5913003] Sastre Pablo, Lobo Jorge M. (2009). Taxonomist survey biases and the unveiling of biodiversity patterns. Biological Conservation.

[B5913077] Vilela Bruno, Villalobos Fabricio (2015). letsR: a new R package for data handling and analysis in macroecology. Methods in Ecology and Evolution.

[B5913086] Waleckx Etienne, Gourbière Sébastien, Dumonteil Eric (2015). Intrusive versus domiciliated triatomines and the challenge of adapting vector control practices against Chagas disease. Memórias do Instituto Oswaldo Cruz.

[B5908833] Organization World Health (2017). Fourth WHO Report on neglected Tropical Diseases: Integrating neglected tropical diseases into global health and development.. World Health Organization.

