# Supplementary material for: Analysis of Chagas disease vectors occurrence data: the Argentinean triatomine species database
Source: Biodivers Data J. 2020 Nov 12;8:e58076. doi: 10.3897/BDJ.8.e58076 (PMC7679344; doi:10.3897/BDJ.8.e58076)
Supplement: Supplementary material 1 — Presence of each triatomine species in the Argentinean ecoregions. [file bdj-08-e58076-s001.html]

**Presence of each triatomine species in the Argentinean
ecoregions.**

Soledad
Ceccarelli, Agustín Balsalobre, María Eugenia Cano, Delmi Canale, Patricia
Lobbia, Raúl Stariolo, Jorge Eduardo Rabinovich & Gerardo Aníbal Marti

 

**Table S1.** Presence of each triatomine species
in the 15 ecoregions, by pre-2000 and post-2000 periods. The last two lines
indicate the number of species registered in each ecoregion by periods (Nsp), and the last column indicates the
number of ecoregions in which a given species is present in each period (Ne). Question mark (?) represents a
dubious species presence in the ecoregion. DCh= Dry
Chaco, PFS= Paraná Flooded Savannas, Es= Espinal, CM= Campos and Malezales, HA= High Andean, IM= Iberá
Marshes, PPM= Plains and Plateaus Monte, HBM= Hills and Bossoms
Monte, Pa= Pampa, PF= Paranense Forest, PS=
Patagonian Steppe, Pu= Puna, PF= Patagonian Forest, HCh=
Humid Chaco and Yu= Yungas.

|  |  |  |  |  |  |  |  |  |  |  |  |  |  |  |  |  |  |
| --- | --- | --- | --- | --- | --- | --- | --- | --- | --- | --- | --- | --- | --- | --- | --- | --- | --- |
|  |  | DCh | PFS | Es | CM | HA | IM | PPM | HBM | Pa | PF | PS | Pu | PF | HCh | Yu | **Ne** |
| *P. geniculatus* | Pre-2000 | x | x |  |  |  | x |  |  |  | x |  |  |  | x |  | **5** |
|  | Post-2000 |  |  |  |  |  |  |  |  |  |  |  |  |  | x |  | **1** |
| *P. guentheri* | Pre-2000 | x |  | x | x |  | x | x | x | x |  |  | ? |  | x | x | **10** |
|  | Post-2000 | x |  |  |  |  |  |  |  |  |  |  |  |  |  |  | **1** |
| *P. megistus* | Pre-2000 | x |  | x | x |  | x |  |  |  | x |  |  |  |  |  | **5** |
|  | Post-2000 |  |  |  |  |  |  |  |  |  |  |  |  |  |  |  | **0** |
| *P. rufotuberculatus* | Pre-2000 |  |  |  |  |  |  |  |  |  |  |  |  |  |  | x | **1** |
|  | Post-2000 |  |  |  |  |  |  |  |  |  |  |  |  |  |  |  | **0** |
| *Ps. coreodes* | Pre-2000 | x | x | x |  |  | x |  |  |  |  |  |  |  | x | x | **6** |
|  | Post-2000 | x | x | x |  |  |  |  | x |  |  |  |  |  | x |  | **5** |
| *T. breyeri* | Pre-2000 | x |  |  |  |  |  |  | x |  |  |  |  |  |  |  | **2** |
|  | Post-2000 | x |  | ? |  |  |  |  | x |  |  |  |  |  |  |  | **3** |
| *T. delpontei* | Pre-2000 | x |  | x |  |  |  |  | x |  |  |  |  |  | x | x | **5** |
|  | Post-2000 | x | x | x |  |  |  |  |  |  |  |  |  |  | x |  | **4** |
| *T. eratyrusiformis* | Pre-2000 | x |  | x |  | ? |  | x | x |  |  | x |  |  |  |  | **6** |
|  | Post-2000 | x |  |  |  |  |  | x | x |  |  | x |  |  |  | x | **5** |
| *T. garciabesi* | Pre-2000 | x |  | x |  |  |  | x | x |  |  |  |  |  |  | x | **5** |
|  | Post-2000 | x |  |  |  |  |  | x | x |  |  |  |  |  | x |  | **4** |
| *T. garciabesi-*  *T. sordida* | Pre-2000 | x |  |  |  |  |  |  |  |  |  |  |  |  | x | x | **3** |
|  | Post-2000 | x |  |  |  |  |  |  |  |  |  |  |  |  |  |  | **1** |
| *T. guasayana* | Pre-2000 | x |  | x |  |  |  | x | x | x |  |  |  |  | x | x | **7** |
|  | Post-2000 | x |  |  |  |  |  | x | x |  |  |  |  |  | x |  | **4** |
| *T. infestans* | Pre-2000 | x | x | x | x | x | x | x | x | x | x | x | X | x | x | x | **15** |
|  | Post-2000 | x |  | x |  |  | x | x | x | x | x | x | x |  | x | x | **11** |
| *T. limai* | Pre-2000 | x |  |  |  |  |  |  |  |  |  |  |  |  |  |  | **1** |
|  | Post-2000 |  |  |  |  |  |  |  |  |  |  |  |  |  |  |  | **0** |
| *T. patagonica* | Pre-2000 | x | x | x |  |  | x | x | x | x |  | x |  |  | x |  | **9** |
|  | Post-2000 | x |  | x |  |  |  | x |  | x |  | x |  |  |  |  | **5** |
| *T. platensis* | Pre-2000 | x | x | x |  |  | x | x | x | x |  |  |  |  | x | x | **9** |
|  | Post-2000 | x |  | x |  |  | x | x | x | x |  | x |  |  | x |  | **8** |
| *T. rubrofasciata* | Pre-2000 |  |  |  |  |  |  |  |  | ? |  |  |  |  |  |  | **1** |
|  | Post-2000 |  |  |  |  |  |  |  |  |  |  |  |  |  |  |  | **0** |
| *T. rubrovaria* | Pre-2000 | ? | x | x |  |  |  |  |  | x | x |  |  |  |  |  | **5** |
|  | Post-2000 |  |  | x |  |  |  |  |  |  |  |  |  |  |  |  | **1** |
| *T. sordida* | Pre-2000 | x | x | x |  |  | x |  |  | ? | x |  |  |  | x |  | **7** |
|  | Post-2000 | x |  |  |  |  | x |  |  |  | x |  |  |  | x |  | **4** |
| **Nsp** | **Pre-2000** | **16** | **7** | **12** | **3** | **2** | **8** | **7** | **9** | **8** | **5** | **3** | **2** | **1** | **10** | **9** |  |
|  | **Post-2000** | **12** | **2** | **7** | **0** | **0** | **3** | **6** | **7** | **3** | **2** | **4** | **1** | **0** | **8** | **2** |  |
